# Deceptive Pseudotuberculous Presentation of a Pulmonary Lepidic Adenocarcinoma

**DOI:** 10.7759/cureus.80947

**Published:** 2025-03-21

**Authors:** Rachid Benchanna, Mohamed Kaakoua, Mohamed Amine Azami, Salah Bellasri, Hicham Janah, Anas Kherrab, Soufiane Sassi, Amine Benjelloune

**Affiliations:** 1 Department of Pulmonology, Avicenne Military Hospital, Marrakech, MAR; 2 Department of Medical Oncology, Avicenne Military Hospital, Marrakech, MAR; 3 Department of Pathology and Laboratory Medicine, Avicenne Military Hospital, Marrakech, MAR; 4 Department of Radiology, Avicenne Military Hospital, Marrakech, MAR; 5 Department of Radiology, Faculty of Medicine and Pharmacy, Cadi Ayyad University, Marrakech, MAR; 6 Department of Rheumatology, Avicenne Military Hospital, Marrakech, MAR; 7 Department of Respiratory Medicine, Avicenne Military Hospital, Marrakech, MAR

**Keywords:** chemotherapy, condensation, excavation, immunotherapy, lepidic adenocarcinoma

## Abstract

Misleading presentations of lepidic adenocarcinomas (ADC) often lead to diagnostic delays, potentially reducing the chances of curative treatment. We report a case of mucinous lepidic ADC that mimicked pulmonary tuberculosis and was diagnosed at a late stage. The tumor showed no epidermal growth factor receptor (EGFR) expression or anaplastic lymphoma kinase (ALK) rearrangement and was characterized by bilateral pulmonary involvement without distant metastases. Through this case, the authors emphasize the need to consider lepidic ADC in cases of chronic pulmonary consolidation, despite atypical radio-clinical presentations that may be encountered in routine practice.

## Introduction

Lepidic adenocarcinoma (ADC) accounts for more than 30% of primary pulmonary adenocarcinomas. It is characterized by locoregional tumor progression with a low incidence of distant metastases. Compared to other forms of non-small cell lung cancer (NSCLC), its prognosis is generally more favorable. The term 'bronchioloalveolar carcinoma' has been replaced by 'adenocarcinoma in situ' (AIS). This pre-invasive lesion is rare, accounting for approximately 5% of NSCLC cases [1]. Lepidic ADC can be classified as either mucinous or non-mucinous [2]. In the latter case, it is essential to specify the predominant histological component (papillary, micropapillary, acinar, or others), as the prognosis is generally better than that of the mucinous subtype [3].

We report a new case of mucinous lepidic ADC in an elderly patient with a history of pleural tuberculosis. The diagnosis was challenging and delayed due to a persistent pneumonia-like presentation with radiological features suggestive of active tuberculosis.

## Case presentation

A 72-year-old male surgeon with a history of treated and cured pleural tuberculosis in 1972, without a history of smoking or occupational/domestic exposure to risk factors, presented with persistent chronic pulmonary consolidation despite initial management. The symptoms began six months earlier with the gradual onset of a productive cough with mucous expectoration, initially minimal but progressively increasing in volume over the past two months. Over the past month, the patient developed effort dyspnea (mMRC stage II) in an afebrile context, along with a weight loss of 7 kg over six months without anorexia.

**Figure 1:**
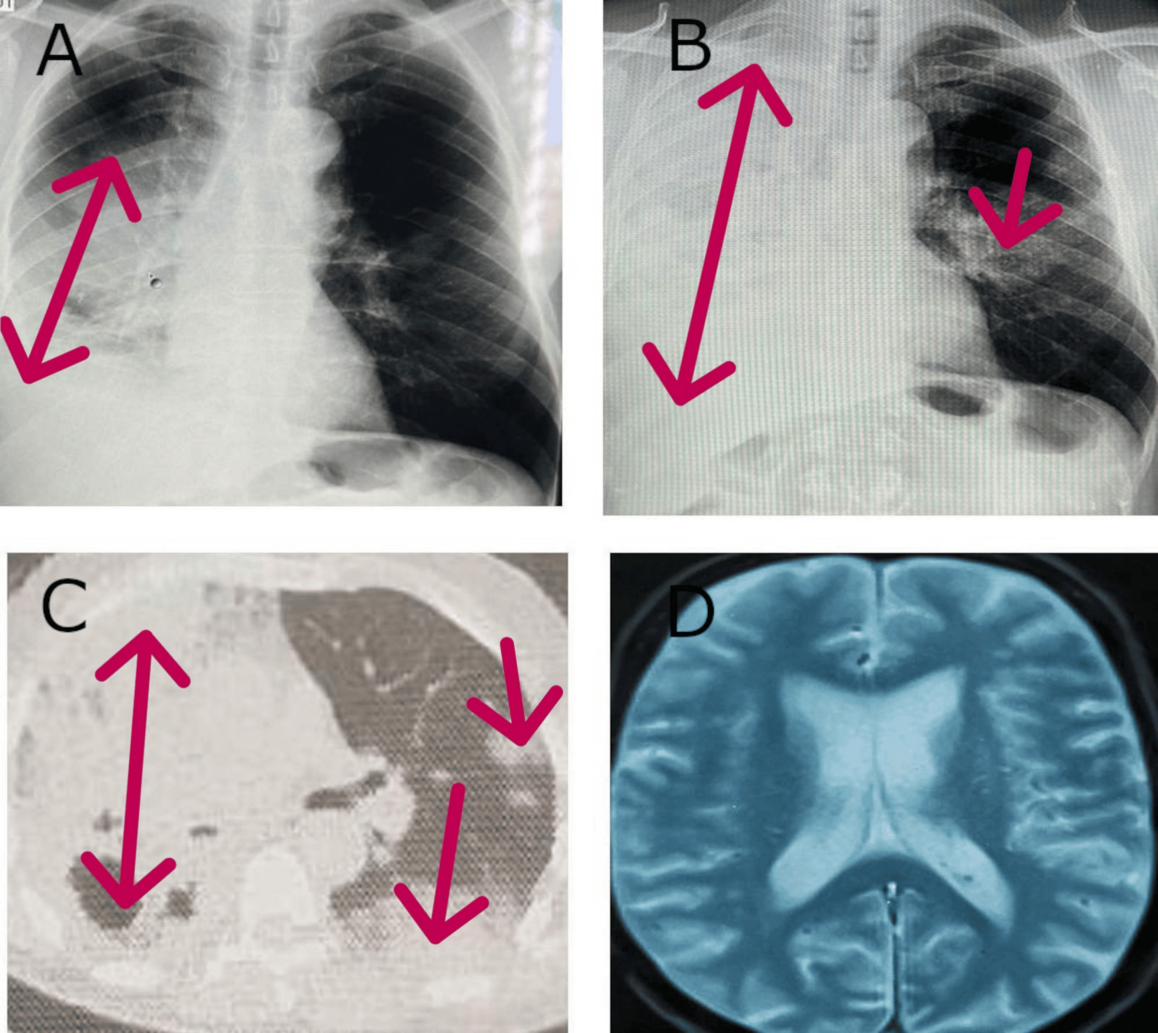
Chest and brain imaging performed. A: Right alveolar opacity associated with minimal pleurisy. B: Opaque hemithorax associated with a contralateral excavated image. C: Right pulmonary condensation with scattered nodules, including one excavated and one in contralateral ground glass. D: Normal cerebral MRI.

**Figure 2:**
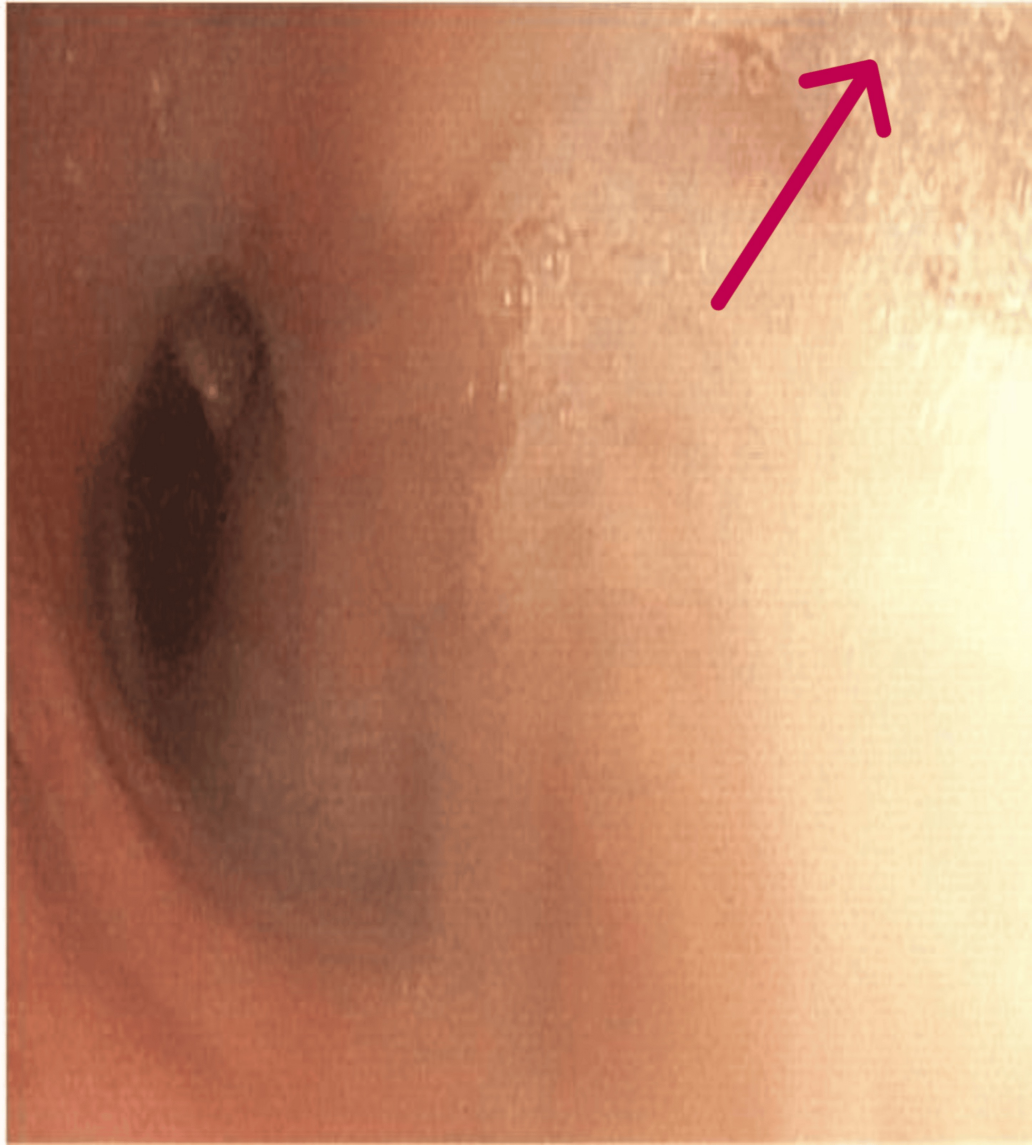
Endobronchial appearance with abundant mucous secretion.

An initial chest X-ray showed alveolar opacity affecting two-thirds of the right lung field, associated with a minimal pleural effusion (Figure 1A). The patient was prescribed amoxicillin/clavulanic acid, followed by endoscopic, biological, and tuberculosis-related investigations. Laboratory results revealed CRP at 25 mg/L (Reference Range (RR) <6 mg/L), WBC counts at 9000/mm³ (RR 4000-9000/mm³), lymphocytes at 1500/mm³ (RR 1500-4000/mm³), and hemoglobin at 14 g/dL (RR 13-18 g/dL). Bronchoscopy showed right ventro-basal spur thickening, with a biopsy revealing nonspecific inflammation and abundant mucous endobronchial secretions (Figure 2). Tests for Mycobacterium tuberculosis in bronchial aspirate, pyogenic bacteria, and Pneumocystis jirovecii were negative.

Due to clinical deterioration despite antibiotic treatment, trimethoprim (160 mg) and sulfamethoxazole (800 mg) were administered three times daily for three weeks without improvement. A follow-up chest X-ray at six months showed complete opacification of the right lung, forming a hemithoracic opaque pattern, along with an excavated opacity in the middle third of the contralateral lung (Figure 1B). Upon consultation, the patient appeared in good general condition, with a BMI of 23 kg/m^2^, stable respiratory and hemodynamic status, and no digital clubbing. Clinical examination revealed right pulmonary consolidation syndrome without other abnormalities.

**Figure 3:**
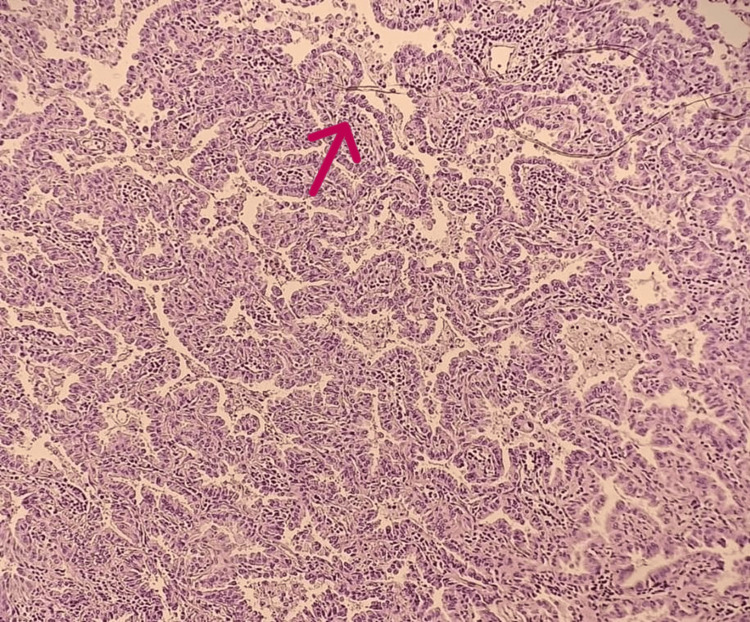
Microscopic image of adenocarcinomatous tumor proliferation showing a lepidic architecture made of tumor cells resembling type 2 pneumocytic cells, growing along the surface of the alveolar spaces (H&E stain, ×200).

A contrast-enhanced and non-contrast chest CT scan revealed consolidation involving the entire right lung parenchyma, associated with cystic images. In the contralateral lung, a suspicious ground-glass excavated lingular nodule was observed (Figure 1C). A CT-guided percutaneous biopsy of the suspicious nodule was performed, and histopathological examination confirmed invasive mucinous lepidic adenocarcinoma (Figure 3). Immunohistochemical staining was positive for Napsin A and CK7, confirming primary pulmonary origin. Oncogenic mutation analysis revealed no EGFR mutation, no ALK rearrangement, and a KRAS gene mutation in codon 12. The PD-L1 expression level was below 1%.

**Figure 4:**
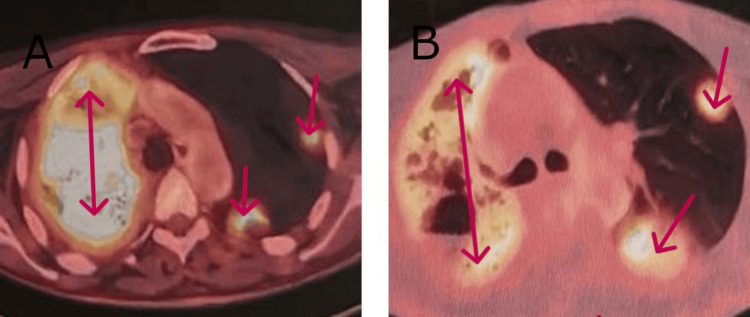
PET scanner requested as part of the extension assessment. A: Hypermetabolism of the entire right lung (9.5 SUV) with sparse nodular involvement contralateral at 7 SUV. B: Reduction in the extent of right lung infiltration; decreased number, size, and uptake of contralateral nodules (2.7 vs. 7 SUV). SUV: Standardized Uptake Value.

A brain MRI (Figure 1D) and PET scan were performed for staging, revealing no extrathoracic or lymphatic metastases. However, diffuse hypermetabolism of the right lung (SUV 9.5) and scattered contralateral nodular involvement (SUV 7) were noted (Figure 4A). A multidisciplinary tumor board decided on three cycles of chemoimmunotherapy with pemetrexed, carboplatin, and pembrolizumab, followed by maintenance therapy with pembrolizumab and pemetrexed every 21 days. At the time of writing, the patient tolerates the prescribed therapy well. A follow-up PET scan showed regression of right lung parenchymal infiltration, with visualization of healthy lung areas and a reduction in the size and number of left lung nodules (Figure 4B).

## Discussion

The classification of ADC has undergone significant changes since 1999. The term 'bronchioloalveolar carcinoma' was abandoned in 2011 due to its heterogeneous prognostic and therapeutic implications [2].

The 2011 IASLC/ATS/ERS classification recognizes three histological types of invasive ADC [3]: (1) ADC with a non-predominant lepidic component (non-mucinous) (formerly mixed ADC); (2) Invasive ADC with predominant lepidic growth (non-mucinous) (formerly bronchioloalveolar component ADC); and (3) Invasive mucinous ADC (formerly mucinous bronchioloalveolar ADC).

The term 'lepidic ADC' encompasses the latter two categories. Bronchioloalveolar carcinoma was previously reserved for forms maintaining pulmonary architecture with lepidic growth without stromal, vascular, or pleural invasion. If invasion was present, the lesion was classified as ADC with a specific histological subtype, such as bronchioloalveolar-component ADC (ADC-CBA) [4]. In 2004, a continuum between CBA and ADC-CBA was demonstrated, leading to the abandonment of the CBA term and its replacement with AIS. A new pre-invasive lesion termed minimally invasive adenocarcinoma (MIA) was also defined. The prognosis of pre-invasive lesions is excellent if completely resected, with a 5-year survival rate of 100% [3].

Our patient presented with invasive mucinous ADC, a subtype characterized by bronchiolar cell metaplasia with excessive mucus secretion. Its prognosis is less favorable than non-mucinous invasive lepidic ADC and is less frequently associated with EGFR amplification/mutation. Instead, KRAS proto-oncogene mutations, as seen in our patient, are more common [5]. Clinically, mucinous ADC often presents with significant bronchorrhea and progressive dyspnea, potentially leading to chronic respiratory failure [6]. Imaging findings range from chronic pulmonary consolidation to multiple nodules with or without ground-glass opacities [7].

The originality of our case lies in its misleading clinical and epidemiological presentation, resembling tuberculosis and leading to a diagnostic delay. Moreover, imaging findings exhibited both a pneumonic and multiple nodular pattern, with an unusual cavitated aspect suggestive of a tuberculosis cavity, which is rare in lepidic ADC. Lepidic ADC is more common in young Asian women and non-smokers [5]. Our case is unique due to the patient’s advanced age at diagnosis.

The treatment of lepidic ADC in locally advanced or contralaterally disseminated forms is superior to other NSCLC subtypes, particularly when the lepidic component is predominant. Non-mucinous forms often express oncogenic markers, making them suitable for targeted therapy [4]. For patients with EGFR mutations, ALK rearrangements, or ROS1 mutations, first-line targeted therapy is indicated [8]. EGFR mutations can be targeted with multiple generations of inhibitors, from first-generation (erlotinib, gefitinib) to third-generation (osimertinib). The FLAURA trial demonstrated that osimertinib significantly improved progression-free survival (18.9 vs. 10.2 months) and overall survival (38.6 vs. 31.8 months) compared to first-generation inhibitors [9]. In the absence of actionable molecular alterations, as in our patient, and with PD-L1 expression >50%, pembrolizumab monotherapy is indicated as first-line treatment. Regardless of PD-L1 status, a combination of pembrolizumab, pemetrexed, and carboplatin for four cycles, followed by pembrolizumab/pemetrexed maintenance, is recommended for two years [8]. A review by Glanville AR and Wilson BE (2018) suggested that extensive lepidic ADC involving both lungs may be eligible for lung transplantation if all cancer treatments have failed and there is no lymphatic or extrathoracic spread [10].

## Conclusions

The revised classification of invasive pulmonary ADC distinguishes non-mucinous forms, which require specification of lepidic predominance, from mucinous lepidic forms. Non-mucinous lepidic ADC has a better prognosis than other histological subtypes. The deceptive presentation of lepidic ADC, as in our case, may lead to delayed treatment, potentially exceeding the window for curative treatment. We emphasize the need for histological confirmation in any chronic, non-resolving pulmonary consolidation, even when an initial diagnosis of infectious pneumonia has been made.
